# The Differential Effects of Good Luck Belief on Cognitive Performance in Boys and Girls

**DOI:** 10.5964/ejop.v15i1.1697

**Published:** 2019-02-28

**Authors:** Lenka Kostovičová

**Affiliations:** a Institute of Applied Psychology, Faculty of Social and Economic Sciences, Comenius University, Bratislava, Slovakia; University of Belgrade, Belgrade, Serbia

**Keywords:** luck-related superstitions, cognitive performance, gender differences, self-efficacy

## Abstract

There is evidence that inducing a luck-related superstition leads to better performance on a variety of motor dexterity and cognitive tasks. However, some replication efforts have failed to succeed. At the same time, our previous findings suggest that the effect of good luck belief on cognitive performance interacts with gender. The present research aimed at replicating the study with a sample of adolescents among whom the superstitious beliefs are particularly prevalent. The participants (N = 99) were allocated to either a control or experimental group, and were asked to solve eight problems focused on cognitive reflection, conjunction fallacy, denominator neglect, and probabilistic reasoning. The experimental manipulation negatively affected boys' performance. Yet, it facilitated performance in girls via increase in their self-efficacy, measured as subjective estimate of future success in the tasks. Thus, gender seems to moderate the effect of luck-related belief on solutions to cognitive problems, which are an important part of our day-to-day decisions. Given initial gender gap in the present tasks, the crucial question to be addressed in future research is possibility of gender being a proxy for prior competence. It would imply that good luck beliefs might help low scorers, for instance in becoming less anxious and more confident, but could be harmful for high scorers.

People tend to believe in things and phenomena despite a lack of empirical support or even when these beliefs are contradicted by evidence (Baron, 2008; Nyhan & Reifler, 2010). Typical examples include pseudoscientific, conspiracy and paranormal claims, whereas believers in one category are susceptible to believe in other categories as well (Lobato, Mendoza, Sims, & Chin, 2014). Beliefs are an important part of human cognition. If one's beliefs are incongruent with aspects of the real world, they are considered a part of the 'contaminated mindware', which leads to maladaptive choices and behaviour (Stanovich, 2009). A special category of these unfounded beliefs is that of superstitions.

In this study I focused on luck-related superstitions. For example, widespread superstitions assign good luck to a four-leaf clover and bad luck to a black cat. Many people have their lucky charms, numbers, rituals or even clothes. Serena Williams, one of the best tennis players, for instance, is known for bouncing the ball five times before the first serve, tying her shoelaces in a specific way, and not changing her socks until a tournament is over (e.g. BBC, 2017). Is she successful despite her epistemically suspect beliefs or thanks to them?

Several authors have suggested that some luck-related superstitions might be beneficial to people. They have found links between belief in good luck and confidence, control and optimism (Darke & Freedman, 1997), positive goal-oriented behaviour (Day & Maltby, 2005), and achievement motivation (Young, Chen, & Morris, 2009). In a baseline study, inducing superstitions associated with luck enhanced performance in motor dexterity, memory and anagram tasks (Damisch, Stoberock, & Mussweiler, 2010). The authors also identified mediators of these effects: increased *self-efficacy* (measured with a 5-item questionnaire), setting *higher goals* (numbers of solutions one aspired to provide in the anagram task) and greater *task persistence* (measured in seconds).

Their paper became a sensation and was extensively addressed by the media. However, replications of one of the experiments failed to succeed (the golfing task; Calin-Jageman & Caldwell, 2014). A meta-analysis (four original experiments, two experiments by the authors, and five more) showed significant heterogeneity in the impact of the superstition on performance. Experimental manipulations in the 11 studies were based on a ball labelled as lucky, a golfer's putter labelled as professional, wishing good luck, bringing one's own lucky charm, subliminal priming, and praying for success (for details on the individual studies, see Calin-Jageman & Caldwell, 2014).

The baseline study led us to a speculation, whether we could, paradoxically, enhance rationality via beliefs that are considered irrational or at least epistemically suspect. Therefore we conducted a study (Kostovičová, Dudeková, & Konečný, 2017) to test the effect of luck-related superstition on performance in cognitive tasks. The superstition was associated with a widely spread symbol of luck - number seven. The cognitive task inventory focused on essential components of informed, rational decisions in everyday functioning, such as probabilistic reasoning and cognitive reflection. In an adult population, we found that fostering luck-related superstition increased self-efficacy (i.e. subjective expected performance) but did not affect the actual performance. However, examining gender differences, we found that our manipulation significantly helped women, yet men's performance remained unaffected. Given almost identical levels of prior belief in luck-related symbols among men and women in our sample, we cannot attribute this finding to gender differences in susceptibility to epistemically suspect beliefs.

However, the tasks used in our study were mainly logical and numerical. Girls and women lack confidence in these types of problems and perceive themselves to be less competent (Goetz, Bieg, Lüdtke, Pekrun, & Hall, 2013), while boys and men tend to have more positive math attitudes (Else-Quest, Hyde, & Linn, 2010) and a lower level of math anxiety (OECD, 2015). Indeed, studies consistently show that men outperform women in numeracy and cognitive reflection (e.g. Campitelli & Gerrans, 2014; Frederick, 2005; OECD, 2015). It is possible that engagement in superstitions related to good luck allows women to manage difficult and challenging situations, such as solving cognitive tasks. Moreover, given low scores of women in the control group, there was much larger room for improvement in the experimental group compared with male participants.

## Research Aim and Hypotheses

In the present research I decided to replicate the study with three important modifications. First of all, I focused on *adolescents*. Damisch et al. (2010) mentioned a high prevalence of superstitious beliefs among sportsmen and students, since they frequently engage in performance tasks. Studying epistemically suspect beliefs among adolescents is particularly important in the context of their scientific literacy as a necessary precondition of a responsible citizenship and informed decisions (Holbrook & Rannikmae, 2007). Indeed, poor results of Slovak high school students in scientific reasoning are a cause for concern (e.g., Čavojová, 2016). Next, in our previous study members of the experimental group were informed on the nature and hypothetical power of the lucky symbol, and the control group proceeded to the cognitive tasks without any specific information. Here I decided for stimuli of an equal length, yet with a different content - so the *cognitive load* is the same in both groups (more details in the Method section). I used a neutral symbol to avoid eventual contamination of the experiment as a result of looking into a classmate´s questionnaire. And finally, experimental material concerned the same cognitive abilities as in the previous study but I chose problems of a higher *ecological validity* given specificities of the current sample. For instance, I replaced the tasks from financial domain with tasks from sport and health domains.

The aim of the present study was to test the effect of inducing belief in good luck on self-efficacy and cognitive performance in adolescents. Based on previous studies, I anticipated an increase in subjective expected performance after inducing superstition related to a symbol ostensibly bringing luck. The main question was whether this induction would have a different impact on boys versus girls in terms of their anticipated and actual performance. Taken together, I was interested in self-efficacy as a mediator of the effect of inducing good luck belief and gender as its moderator.

## Method

### Participants and Design

I assumed similar effects to that in our previous study (Kostovičová et al., 2017; *d* = 0.57) and α = .05, 1 – β = .80, and two-sided tests. Therefore, I planned to recruit 50 participants per experimental conditions. I recruited 110 adolescents from one state secondary school in Slovakia. Eleven students were excluded due to missing informed consent signed by one of their parents. The final sample consisted of 39 boys and 60 girls, aged 15 to 18 years (*M* = 16.8, *SD* = 1.0). Data collection in a pen-and-paper form was conducted in four sessions on the same day. There were no financial incentives included.

The participants were randomly allocated to one of the two conditions: the control group without induction (*n* = 49) or the experimental group with induction of the luck-related superstition (*n* = 50). Experimental manipulation was based on the presence of a symbol, which was located at the top of a paper with the tasks. The control group was informed that it is a symbol of life, representing that everything in our lives is ambiguous, interrelated, and that life is a repeating cycle. The experimental group was told that it is a lucky symbol, believed by many to bring luck, happiness and success, and that it might help them, too. This description was the only difference in the materials provided to the two groups.

### Procedure and Materials

Among other instructions, the participants were told that they would solve eight word problems focused on thinking and reasoning, of varying difficulty. The instructions were followed by information on what the symbol in the right corner of the paper stands for, as described in the previous section. A control question was included here as an attention check to ensure that the participants did not skip this crucial part of the experimental material [“Have you seen this symbol before?” (no/yes)]. Subsequently, the participants were asked to rate their subjective expected performance and, thus, their perceived self-efficacy [“Please make an estimate on how many of the eight tasks you will solve correctly:” (0–8)].

Afterwards, they solved two verbal cognitive reflection problems (*Daughters*, *Train*), two numerical cognitive reflection problems (*Pig*, *Barrel*), one conjunction fallacy task (*Volleyball*), one denominator neglect task (*Drug*), one conditional probability problem (*Disease*) and one cumulative probability problem (*Pregnancy*). Listed below are the problems in original English wording, without introductory sentences, with correct/rational responses (CR) and intuitive responses (IR) in parentheses. Accuracy of the Slovak wording was ensured with back-translation.

*Daughters*: “Mary’s father has 5 daughters but no sons – Nana, Nene, Nini, Nono. What is the fifth daughter's name probably?” (Sirota, Kostovičová, Juanchich, Dewberry, & Marshall, 2018; CR: Mary, IR: Nunu)

*Train*: “The wind blows west. An electric train runs east. In which cardinal direction does the smoke from the locomotive blow?” (Sirota et al., 2018; CR: no smoke / no direction, IR: west)

*Pig*: “A man buys a pig for $60, sells it for $70, buys it back for $80, and sells it finally for $90. How much has he made?” (Toplak et al., 2014, p. 151; CR: $20, IR: $10)

*Barrel*: “If John can drink one barrel of water in 6 days, and Mary can drink one barrel of water in 12 days, how long would it take them to drink one barrel of water together?” (Toplak, West, & Stanovich, 2014, p. 151; CR: 4 days, IR: 9 days)

*Volleyball*: “…Volleyball players have grown younger yet taller. Women players are on average taller than 1.80 m, ranging between 1.75 m for some setters to more than 1.90 m for many spikers. Suppose we choose at random 100 female volleyball players. Which group do you think is the most numerous? a. Women who are less than 21 years old, b. Women who are less than 21 years old and are taller than 1.77 m, c. Women who are less than 21 years old and are not taller than 1.77 m” (Tentori, Bonini, & Osherson, 2004, p. 474; CR: a, IR: b)

*Drug*: “… A study with 900 with high cholesterol showed that 80 of the 800 people who have not taken the drug deceased after a heart attack, compared with 16 of the 100 people who have taken the drug. How beneficial was the Benofreno? Not beneficial 1 2 3 4 5 6 7 Very beneficial” (Ghazal, Cokely, & Garcia-Retamero, 2014, p. 33; CR: 1, IR: 7)

*Disease*: “… A person has a 6 out of 100 chance of having the infection. If a person is infected with Disease X, then the chance that he will have a positive result in the test is 4 out of the 6. On the other hand, if a person is not infected with Disease, then the chance that his test is also positive is 16 out of the 94. Imagine a common European is tested now. His test turns out to be positive. What is the chance that he is really infected with Disease X? ____ out of ____” (Sirota, Kostovičová, & Juanchich, 2014, p. 373; CR: 4 out of 20, IR: 6 out of 100)

*Pregnancy*: “The probability that a woman using the contraceptive Primon will experience no unwanted pregnancies at all during a period of one year is 90%. What is the probability that she will experience no unwanted pregnancies at all in a three-year period? (McCloy, Byrne, & Johnson-Laird, 2010, p. 515; CR: 72.9%, IR: 90%)

### Measures and Reliability

It is important to distinguish between the intuitive and other incorrect responses. A person might overcome initial automatic responses (i.e. error detection), yet might not be able to come up with a correct solution (i.e. error correction). Thus, intuitive responses score stands for *intuition inhibition failure* and correct responses score stands for *analytic thinking ability*, often used as a synonym for rational thinking.

As reported in more detail within the following section, the participants failed to solve the conditional and cumulative probability problems. Since reliability of the cognitive performance measure was low (Cronbach's α = .52), I conducted a basic exploratory factor analysis which resulted in two principal components: cognitive reflection (CRT) and probabilistic reasoning (PR). The components were positively correlated, *r* = .30, *p* = .002. Therefore I run the analyses with the composite scores and the two separate scores as well.

## Results

### The Effects of Experimental Manipulation on Performance

Percentages of correct and intuitive answers in the two groups and overall are provided in Table 1. Success rate in the cognitive reflection tasks varied between 27 and 64 percent. The probabilistic reasoning problems proved to be difficult for the participants. Less than a third managed to provide correct responses to the denominator neglect and conjunction fallacy tasks, and none of them were able to solve the conditional and cumulative probability problems.

**Table 1:** Correct and Intuitive Responses in the Eight Tasks (%)

Task	Control group		Experimental group		Overall	
	Correct	Intuitive	Correct	Intuitive	Correct	Intuitive
Daughters	59.2	38.8	66.0	34.0	63.6	36.4
Train	42.9	57.1	48.0	50.0	45.5	53.5
Pig	32.7	51.0	42.0	42.0	37.4	46.5
Barrel	28.6	26.5	26.0	24.0	27.3	25.3
Volleyball	24.5	73.5	34.0	60.0	29.3	66.7
Drug	18.4	4.1	10.0	2.0	14.1	3.0
Disease	0	4.1	0	20.0	0	12.1
Pregnancy	0	63.3	0	80.0	0	71.7

Moreover, while 92% of the participants expected a score above 3 out of 8 points (*M* = 4.6, *SD* = 1.7), their success rate was much lower — only 12% correctly solved more than three tasks (*M* = 2.2, *SD* = 1.3). On the other hand, almost all participants (93%) provided at least two intuitive responses (*M* = 3.1, *SD* = 1.2). The correlation between the estimate and the performance was weak, *r* = .28, *p* = .006, as was the negative link between the estimate and the intuitive responses, *r* = -.22, *p* = .032. Rational and automatic intuitive responses were strongly negatively related, *r* = -.70, *p* < .001.

Analyses of individual tasks showed no significant differences between the control and experimental group in the proportion of correct answers. As for the intuitive responses, they were more frequent in the experimental group in the case of the Disease problem, χ^2^(1) = 5.59, *p* = .018, φ = 0.24.

**Figure 1:**
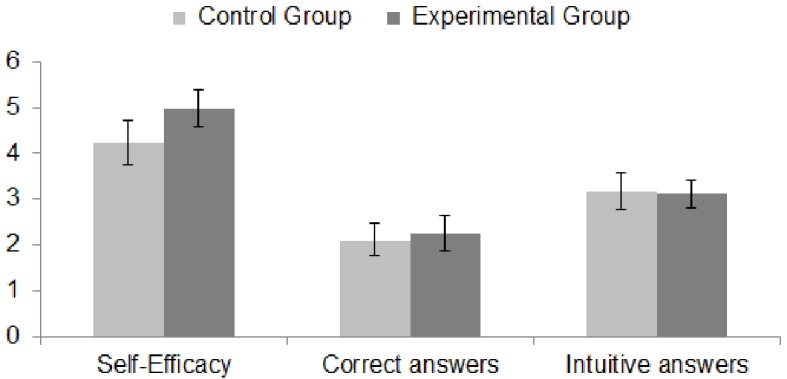
Comparisons between the control group and the experimental group.

Differences between the two groups in self-efficacy (0–8), the correct responses score (0–6) and the intuitive answers score (0–6) are depicted in Figure 1. Fostering luck-related superstition increased perceived self-efficacy, *t*(97) = -2.23, *p* = .028, *d* = 0.45, but did not affect the performance in terms of either rational responses, *t*(97) = -0.62, *p* = .540, *d* = 0.12, or intuitive answers, *t*(97) = 0.17, *p* = .862, *d* = 0.03. No significant effect of good luck belief was found with respect to the two distinct components (CRT & PR). Despite the fact that examining mutual patterns in the situation of non-significant total effects may bring interesting insights into underlying mechanisms (e.g., Hayes, 2009), I did not proceed with analysing the indirect effects of manipulation on performance via self-efficacy.

### The Differential Effects of Experimental Manipulation by Gender

Overall gender comparisons in the control and the experimental group are depicted in Table 2. In the control group, boys expressed greater self-efficacy, *t*(47) = 2.54, *p* = .015, *d* = 0.80, and provided more correct answers, *t*(47) = 3.93, *p* < .001, *d* = 1.24, and less intuitive answers, *t*(47) = -2.77, *p* = .008, *d* = 0.87, than did girls. Yet, there were no gender differences in the experimental group (*p* > .05, *d* < 0.20).

**Table 2:** Comparisons Between Boys and Girls – Composite Scores

Expected and actual performance	Control group				Experimental group			
	Boys		Girls		Boys		Girls	
	*M*	*SD*	*M*	*SD*	*M*	*SD*	*M*	*SD*
Self-efficacy	5.2	2.4	3.9	1.3	5.1	1.4	4.8	1.6
Correct answers	3.1	1.3	1.7	1.0	2.2	1.5	2.4	1.1
Intuitive answers	2.4	1.2	3.5	1.3	3.1	1.1	3.1	1.1

Partial gender comparisons are depicted in Table 3. In the control group, boys performed better in cognitive reflection tasks (CRT), *t*(47) = 4.13, *p* < .001, *d* = 1.31, and provided less intuitive CRT answers, *t*(47) = -3.68, *p* = .001, *d* = 1.16, than did girls. I failed to find any substantial differences with respect to the probabilistic reasoning (PR) accuracy. In the experimental group, there were no gender differences at all (*p* > .05, *d* < 0.20).

**Table 3:** Comparisons Between Boys and Girls – Partial Scores

Cognitive performance	Control group				Experimental group			
	Boys		Girls		Boys		Girls	
	*M*	*SD*	*M*	*SD*	*M*	*SD*	*M*	*SD*
Correct responses								
CRT	2.5	0.9	1.3	0.9	1.8	1.2	1.8	1.0
PR	0.6	0.8	0.4	0.5	0.3	0.6	0.6	0.6
Intuitive responses								
CRT	0.9	0.9	2.1	1.1	1.5	1.0	1.5	0.9
PR	1.5	0.8	1.4	0.7	1.6	0.7	1.6	0.7

Moreover, the manipulation had negative effects on boys: it decreased their performance (*M*_DIFF_ = -0.9), *t*(37) = 1.90, *p* = .065, *d* = 0.63, and amplified the intuitiveness of their answers (*M*_DIFF_ = 0.8), *t*(37) = -2.05, *p* = .047, *d* = 0.68. It did not affect their self-efficacy (*M*_DIFF_ = -0.1), *t*(37) = 0.16, *p* = .877, *d* = 0.05. The partial effects on cognitive reflection were moderate and marginally significant, correct answers (*M*_DIFF_ = -0.7): *t*(37) = 1.92, *p* = .063, *d* = 0.64; intuitive answers (*M*_DIFF_ = 0.6): *t*(37) = -2.03, *p* = .050, *d* = 0.68, and the effect on probabilistic reasoning was small and non-significant (*M*_DIFF_ = -0.3), *t*(37) = 1.12, *p* = .272, *d* = 0.37.

In contrast, the manipulation had positive effects on girls: it boosted their self-efficacy (*M*_DIFF_ = 1.0), *t*(58) = -2.59, *p* = .012, *d* = 0.68, and facilitated their performance (*M*_DIFF_ = 0.7), *t*(58) = -2.37, *p* = .021, *d* = 0.62. It slightly, albeit non-significantly, decreased the intuitiveness of their answers (*M*_DIFF_ = -0.4), *t*(58) = 1.13, *p* = .265, *d* = 0.29. The partial effects on cognitive reflection were moderate and significant, correct answers (*M*_DIFF_ = 0.5): *t*(58) = -2.05, *p* = .045, *d* = 0.54; intuitive answers (*M*_DIFF_ = -0.6): *t*(58) = 2.11, *p* = .039, *d* = 0.55, and the effect on probabilistic reasoning was small and non-significant (*M*_DIFF_ = 0.2), *t*(58) = -1.28, *p* = .205, *d* = 0.34.

**Figure 2:**
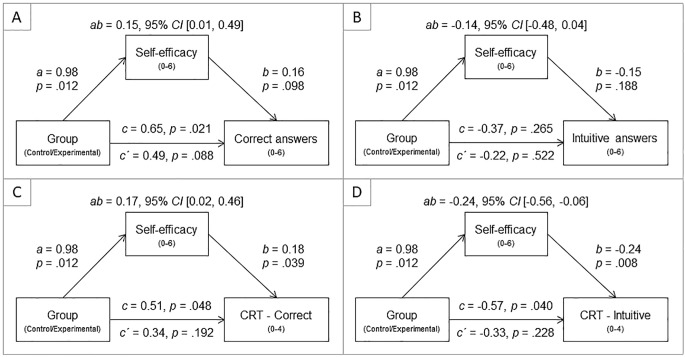
Mediation models – Girls only.

Finally, based on the observed patterns, I conducted mediation analyses so as to test the effects of manipulation on performance via self-efficacy separately in girls. The analysis was conducted using the SPSS macro PROCESS (Hayes, 2013; 10,000 bootstrap samples). I tested four models as depicted in Figure 2, with overall performance - correct responses (Model A), overall performance - intuitive responses (Model B), correct CRT responses (Model C) and intuitive CRT responses (Model D) as dependent variables. Experimental manipulation boosted self-efficacy (*a* path), and self-efficacy had a substantial effect on CRT answers but not on overall responses (*b* paths). All total effects (*c* paths) and indirect effects (*ab* paths) were significant, except for model B with overall intuitive answers. All direct effects (*c´* path) were non-significant. In sum, self-efficacy mediated the effect of good luck belief on overall cognitive performance, and particularly on error detection and error correction in the cognitive reflection tasks.

## Discussion

In the present study I addressed a somewhat controversial question regarding the positive effect of luck-related superstitions on thinking and reasoning. In their widely discussed paper entitled “Keep Your Fingers Crossed! How Superstition Improves Performance”, Damisch et al. (2010) demonstrated performance benefits of this specific category of beliefs in golfing, motor dexterity, memory, and anagram tasks. However, a high-powered follow-up replication with the golfing task (Calin-Jageman & Caldwell, 2014) failed to support the original findings, and the authors speculated about some possible unknown moderator.

In line with the previous study (Kostovičová et al., 2017), I found that fostering belief in good luck helped girls through encouraging their self-efficacy. Surprisingly, this induction was not beneficial to boys — it tended to impair their success in solving cognitive tasks. In the two successful experiments with cognitive tasks by Damisch et al. (2010), the vast majority of participants were female (80% and 87%, respectively).

Mediation analysis showed that the experimental manipulation worked because it helped girls to become more self-efficient. An interesting issue is whether this could be a general pattern or it only works for specific problems and conditions. The cognitive tasks employed in this study were mainly numerical and logical. Gender gap in perceived competency, confidence, attitudes, anxiety and actual performance in this domain has been repeatedly documented (e.g., Campitelli & Gerrans, 2014; Else-Quest et al., 2010; Frederick, 2005; Goetz et al., 2013; OECD, 2015). Inducing luck-related superstition might mitigate the gap thanks to supporting self-confidence and task persistence in female participants. In addition, problems associated with analytic thinking are likely to be especially difficult due to gender stereotypes and stereotype threat. Thus, if negative stereotypes exist regarding a specific group, its members are prone to become anxious about their performance, which may impede the ability to take advantage of one's own potential (Steele & Aronson, 1995). Besides physiological stress, stereotype threats also seem to consume executive capacity through actively monitoring own performance, and suppressing negative emotions and thoughts (Schmader, Johns, & Forbes, 2008). Encouragement in a form of luck-related superstition might help to counteract these processes.

On the other hand, the drop in boys' performance might be caused by unfavourably affecting those attributes that are already close to being optimal (e.g., perceived competence). This might lead to underestimation of task difficulty and less motivation and effort. In order to test this assumption, as well as other possible explanations, further research is necessary. It would be interesting to examine the effect of inducing luck-related superstition in domains wherein women usually exhibit higher performance than men (e.g. recognising emotions: Donges, Kersting, & Suslow, 2012) or in domains without gender differences (e.g. verbal abilities: Wallentin, 2009). But most importantly, we need to investigate whether gender is only a proxy for prior competence. This would imply that the effect of good luck belief is beneficial to initial low scorers but harmful to initial high scorers — at least in some specific tasks.

The reasons for different findings from those of some studies in the meta-analysis might undoubtedly be the material and the manipulation used. Both the cognitive problems and the induction method were novel and their link to the effect of good luck belief has not been tested by other researchers. Nevertheless, all of the previous studies, except for the direct replication, are very diverse in terms of the manipulation and the tasks. Fostering luck-related superstitions ranged from praying for success (Aruguete, Goodboy, Jenkins, Mansson, & McCutcheon, 2012) to a ball labelled as lucky by the experimenter (Calin-Jageman & Caldwell, 2014; Damisch et al., 2010). Similarly, there was a high level of heterogeneity in the dependent measures, with the golf task being the most common one. Cognitive reflection and other competencies that were part of the present research play an important role in everyday problem solving and decision making (e.g. Garcia-Retamero, Galesic, & Dhami, 2013; Juanchich, Dewberry, Sirota, & Narendran, 2016; Toplak, West, & Stanovich, 2011). Finding ways in which to improve them is, therefore, of high relevance, as is the need for replications in experimental psychology (Francis, 2012). Another advantage of our approach is methodological rigor so that the results are not affected by the experimenter (who wishes good luck), guessing the purpose of the study (when being told that the ball is lucky) or recruiting believers only (who can bring their own lucky charm).

Future studies could address some limitations of the present research. First of all, other measures of rationality should be considered, since the present tasks do not cover all its important components, and two tasks proved to be too difficult for the participants. Next, I did not include manipulation check because I assumed that it might contaminate the study by, for instance, drawing people’s attention to the purpose of the research. Moreover, in contrast with our previous study, I found the effect of luck-related belief on cognitive reflection but not on probabilistic reasoning. For this and other reasons, direct replications as well as conceptual replications with different cognitive tasks are needed.

If beauty is in the eye of the beholder, maybe the same applies to rationality. One can say that faith in lucky objects and symbols is epistemically suspect and unjustified. However, if using a pen perceived to be lucky helps people to be more relaxed, optimistic, confident and persistent (and sometimes even more successful), then the dispute surrounding the rationality of this strategy is far from being finished. Under certain circumstances, paradoxically, fostering epistemically suspect beliefs may stimulate rationality.
